# Potential roles of microRNAs in regulating long intergenic noncoding RNAs

**DOI:** 10.1186/1755-8794-6-S1-S7

**Published:** 2013-01-23

**Authors:** Liran Juan, Guohua Wang, Milan Radovich, Bryan P Schneider, Susan E Clare, Yadong Wang, Yunlong Liu

**Affiliations:** 1 Center for Biomedical Informatics, Harbin Institute of Technology School of Computer Science and Technology, Harbin, Heilongjiang 150001, China; 2 Department of Surgery, Indiana University School of Medicine, Indianapolis, IN 46202, USA; 3 Department of Medicine, Indiana University School of Medicine, Indianapolis, IN 46202, USA; 4 Department of Medical and Molecular Genetics, Indiana University School of Medicine, Indianapolis, IN 46202, USA; 5 Center for Computational Biology and Bioinformatics, Indiana University School of Medicine, Indianapolis, IN 46202, USA

## Abstract

**Background:**

Over 10,000 long intergenic non-coding RNAs (lincRNAs) have been identified in the human genome. Some have been well characterized and known to participate in various stages of gene regulation. In the post-transcriptional process, another class of well-known small non-coding RNA, or microRNA (miRNA), is very active in inhibiting mRNA. Though similar features between mRNA and lincRNA have been revealed in several recent studies, and a few isolated miRNA-lincRNA relationships have been observed. Despite these advances, the comprehensive miRNA regulation pattern of lincRNA has not been clarified.

**Methods:**

In this study, we investigated the possible interaction between the two classes of non-coding RNAs. Instead of using the existing long non-coding database, we employed an *ab initio *method to annotate lincRNAs expressed in a group of normal breast tissues and breast tumors.

**Results:**

Approximately 90 lincRNAs show strong reverse expression correlation with miRNAs, which have at least one predicted target site presented. These target sites are statistically more conserved than their neighboring genetic regions and other predicted target sites. Several miRNAs that target to these lincRNAs are known to play an essential role in breast cancer.

**Conclusion:**

Similar to inhibiting mRNAs, miRNAs show potential in promoting the degeneration of lincRNAs. Breast-cancer-related miRNAs may influence their target lincRNAs resulting in differential expression in normal and malignant breast tissues. This implies the miRNA regulation of lincRNAs may be involved in the regulatory process in tumor cells.

## Background

Deep sequencing data from the Encyclopedia of DNA Elements Consortium (ENCODE) suggests that 70-80% of the human genome can be transcribed, and non-protein-coding RNAs (ncRNA) exceed the number of protein-coding genes [1]. The recent discovery of a large number of non-coding RNAs (ncRNAs) significantly enriches the portfolio of potential genetic factors. Rather than being transcriptional noise, many ncRNAs serve as master regulators that affect expression levels of dozens or even hundreds of target genes [2-7]. These regulatory RNAs integrate signals from both genetic and environmental factors, and therefore can play major roles in controlling biological processes [8]. Most notably, a strong association of epigenetic marks with long intergenic non-coding RNAs (lincRNAs >200 nucleotides) in humans and mice was recently described [9-11]. The lincRNAs show evolutionary conservation and spatiotemporally restricted expression patterns, implying that they are functional and regulated. These lincRNAs are reported to regulate dosage compensation, imprinting, and development by establishing chromatin domains in an allele- and cell-type specific manner [12,13]. According to an analysis on half-lives, lincRNAs are more stable than intron-derived long noncoding RNAs, as they are usually spliced, which also suggest widespread functionality [14].

In two recent studies, over 10,000 lincRNA regions were identified in human and mouse genomes. These regions were discovered based on epigenetic marks, where the promoter mark H3K4me3 is followed by the transcript mark H3K36me3. Comparative genomics analysis suggests that a significant portion of these regions do not have strong protein coding potentials, and do not have sequence homology with other known proteins [2]. The K4-K36 signature indicates that the transcription of these non-coding RNAs is regulated in a similar fashion as protein-coding genes. Although the molecular mechanisms through which most lincRNAs function were unknown, diverse regulatory mechanisms have been reported for several well-characterized lincRNAs [15,16]. By targeting the chromatin modification complexes and RNA-binding proteins (XIST, AIR) [17,18], they can inhibit gene expression (HOTAIR, DBE-T) [12,19], and control alternative splicing (MALAT-1) [20]. In addition, some lincRNAs are found to be differentially expressed (HOX) or coactivate other proteins (SRA) in tumor cells [10,21], and are reported to be strongly associated with tumorigenesis [22,23].

MicroRNA (miRNA) is another type of noncoding RNA whose biological functions have been extensively studied. MiRNAs are ~22nt small non-coding RNAs [24] that promote mRNA degeneration and/or inhibit their translation by complementarily binding on the 3' un-translated region (3'UTR) of mRNAs [25] and attracting RNA-induced silencing complex (RISC). In the past decades, over one thousand miRNAs have been identified [25,26]. They are broadly related with regular cell functions and diseases [27-30].

Despite the extensive investigation on the roles of miRNAs in regulating protein-coding genes, only a few isolated non-coding transcripts (CDR1 antisense and MEG3) are reported to be regulated by miRNA [31,32]. Though CDR1 antisense is not an intergenic ncRNA, and MEG3 is indirectly regulated by miR-29, these discoveries encouraged us to explore the potential roles of miRNA in regulating non-specific lincRNA expression. Because the primary mechanism of miRNA regulation is by targeting RNA for degradation [33], they are likely to also broadly regulate lincRNA expression. In fact, 5% of genomic regions interacting with argonaute proteins are located in non-coding RNAs [34]. The argonaute protein family is one of the major components of the RNA-induced silencing complex (RISC) that is responsible for gene silencing due to miRNA expression and RNA interference [35]. In this study, we intend to generally investigate the potential roles of miRNAs in regulating the lincRNA expression.

GENCODE project and NONCODE database have both collected and integrated long noncoding RNA annotations[36,37]. Based on GENCODE annotation, miRcode provides a map of possible miRNA targets on long noncoding RNAs by using multiple bioinformatics prediction software [38]. However, in tissue-specific lincRNA studies, the *ab initio *approach is usually employed to reconstruct the annotation of lincRNAs from the RNA-seq data [2,3,39]. Considering that lincRNA shows a high tissue or cell-specific expression pattern [3], this approach is also appropriate for our study.

RNA-seq experiments sequence millions to billions of short RNA fragments in a single experiment, by which it measures the expression levels of the whole transcriptome, including noncoding RNAs, without relying on detailed annotation. To explore the potential miRNA-lincRNA expression relationship, we analyzed RNA-seq data from a group of normal and malignant breast tissues. Random primers were used in the reverse transcription step, and the strands of the transcripts were preserved to aid in identifying non-coding RNAs that are not poly-adenylated. Due to their small size (~22nt), the mature miRNAs were not included in the sequencing libraries. However, our data suggested that we can detect precursor miRNAs (80-120nt) from these RNA-seq data sets. For lincRNAs, the sequence fragments across two exons of the transcripts (junction reads) are able to reveal exon boundaries, especially novel ones. This feature helps us to understand the splicing patterns of unannotated lincRNAs. Several tools have been developed for mapping junction reads [40], detecting the splicing events, assembling isoforms and estimating expression [41,42].

In this study, for each putative miRNA-lincRNA pair, (1) we required reverse correlation between the expression levels of pre-miRNA and lincRNA within the 20 examined tissue samples. This is due to the well-established roles of miRNAs in facilitating RNA degradation on protein coding genes; and (2) we also required seed sequence of the miRNA (positions 2-7 on the miRNA) should be present in the exon regions of identified lincRNA, and the surrounding sequences on the lincRNA should also favor miRNA targeting conditions. We further examined the evolutionary conservation of the identified miRNA seeds in lincRNAs.

## Results

### Overall strategy

**Figure 1:**
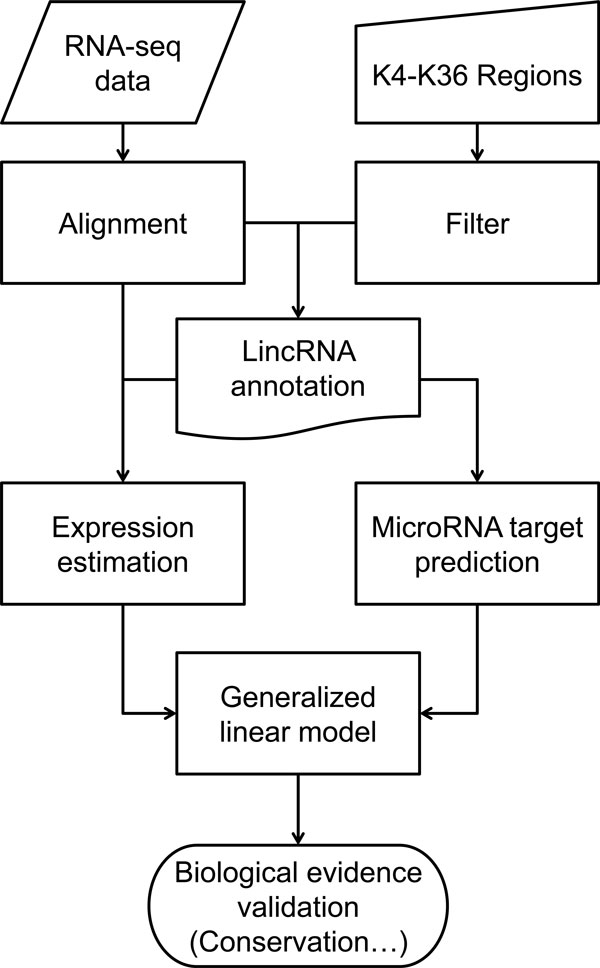
**The schematics of the overall study**.

In order to investigate the potential relationships between miRNA and lincRNA, we used RNA-seq data from 20 samples, including 10 breast tumor samples, and 10 breast tissues from healthy pre-menopausal volunteers with no history of disease; these normal tissues were obtained from the Susan G. Komen for the Cure^® ^Tissue Bank at the IU Simon Cancer Center. As shown in Figure 1, our analysis includes the following steps: (1) Sequence alignment. RNA-seq reads were aligned to the human genome (hg18) and transcriptome using a customized pipeline by a TopHat-like strategy with BFAST as the primary aligner [40,43]. (2) Candidate lincRNA identification. LincRNA identification focuses on the noncoding regions showing a K4-K36 signature from previous studies [2,3]. To ensure the key properties of lincRNAs (intergenic, detectable transcription activities, and no coding potentials), the regions were further filtered by their distance to the nearest known gene, signal levels from RNA Polymerase II (RPolII) ChIP-seq data, and coding substitution frequency (CSF) scores. (3) Reconstructing lincRNA exonic structures. In these candidate lincRNA regions, we reconstructed the lincRNA transcript by searching the potential splicing events supported by RNA-seq novel junction reads around the putative exons. (4) MiRNA binding sites were predicted on lincRNA sequences derived from transcript reconstruction. (5) Expression levels of mRNAs, lincRNAs and miRNAs were estimated based on the number of RNA-seq reads falling into their respective genomic regions. (6) Relationships of the expression levels of the mRNA/lincRNA-miRNA pairs were estimated using a generalized linear model (GLM). (7) The conservation features of the predicted miRNA sites were evaluated on the pairs showed a significant reverse correlation on their expression (Figure 1).

### LincRNA annotation

Based on the RNA-seq data, the discovery of lincRNAs in breast tissue includes the following steps: (1) Epigenetic marks and comparative genetics. As reported previously, thousands of lincRNAs have been discovered by searching for the genomic regions with enhanced promoter marks (H3K4me3, or K4) following the transcript marks (H3K36me3, or K36). Our analysis started from 2,513 intergenic K4-K36 regions in the human genome (hg18) and 1,665 intergenic K4-K36 regions in the mouse genome (mm9) from previous studies [2,3]. We further mapped the mouse intergenic K4-K36 regions to 1,502 genomic loci in human genome using the liftOver tool in the UCSC Genome Browser [44]. LiftOver can convert genomic coordinates between selected assemblies of the same or different species. After merging the two sources of K4-K36 regions and removing the ones near or overlapping with known genes, 3,096 candidate lincRNA regions remained.

**Figure 2:**
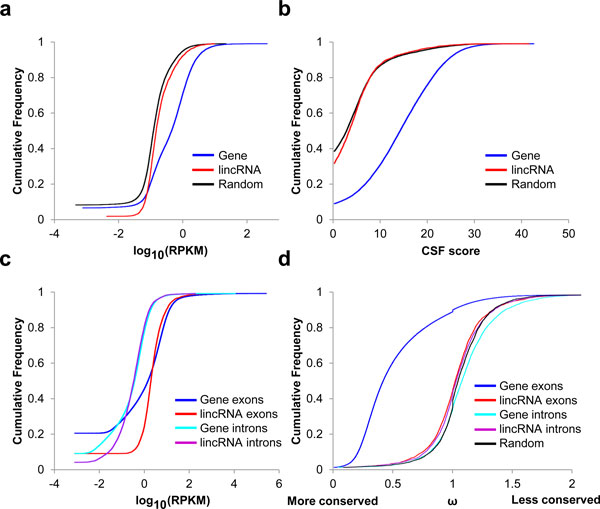
**RPolII signal, CSF scores, expression levels and conservation in lincRNA regions**. (a) The RNA polymerase II ChIP-seq signals in protein coding genes, lincRNA regions, and randomly selected regions. The RPolII signal is demonstrated as reads per kilobase exon model per million mappable reads. (b) The CSF score of the lincRNA regions, protein-coding genes, and randomly selected regions. (c) The RPKM values for the exonic and intronic regions for both lincRNAs and protein-coding genes. (d) Conservation levels for exonic regions, intronic regions for both lincRNAs and protein-coding genes. X axis denotes the conservation score ω  derived from Siphy prediction. A smaller and larger ω  value indicates more and less conserved, respectively.

(2) Evidence of transcriptional activity in other breast cancer cell lines. We have previously reported genome-wide binding patterns of RNA polymerase II (RPolII) in MCF7 cell lines [45], derived from chromatin immunoprecipitation following high throughput sequencing technology (ChIP-seq). Although not directly measure the transcriptional activity for the tissues being measured, the RPolII signals in the MCF7 provide fair estimation in breast cells. We infer the lincRNA transcriptional activity by counting the number of ChIP-seq-derived RPolII tags falling into the lincRNA genomic regions; these counts were further normalized by the length of the lincRNA and the total number of reads for the sample (similar to the RPKM measure in RNA-seq studies [46-48]). Consistent to the results from previous studies [45], the lincRNAs show intermediate transcriptional activities between protein coding genes and random genomic regions (Figure 2a). We focused our further analysis on the 2,120 lincRNAs (out of 3,096) whose RPolII signal intensities were more than 0.1 RPKM.

(3) Coding potential. We evaluated coding potentials of the identified lincRNA regions using the methods presented in [49,50]. By modeling the mammalian Codon Substitution Frequency (CSF) of transcript regions and random genomic regions, a CSF score was calculated for each region to represent the codon substitution pattern of the region. A higher CSF score indicates higher potential for being a protein coding gene. The coding potentials of the lincRNA regions are slightly but significantly higher than the ones in random genomic regions (p < 5.4e-07, Wilcoxon test on CSF scores), while the coding potential of protein-coding genes is much higher than lincRNA regions (Figure 2b). For further analysis, we excluded 47 regions with high CSF scores >20; these regions may represent protein-coding genes that are not included in the current annotation database.

We used Scripture [42] for reconstructing lincRNA exon structures from RNA-seq data. Scripture scans read-enriched regions as putative exons and finds exon boundaries supported by the reads across potential junctions. Scripture identified 525 lincRNAs in the 2,073 candidate regions; this percentage is comparable with the lincRNAs identified by previous studies in other tissues. The genomic coordinates of these lincRNAs are listed in Additional file 1. The average lincRNA length is 1201.7 bases, 75.2% of which are shorter than 1000 bases. The longest lincRNA has 32,178 bases. The lincRNAs are composed by 7.12 exons on average. The mean exon length is 168.8 bases. Almost half (46.7%) of the exons are shorter than 100 bases, with the longest has 5,242 bases. The annotated lincRNAs show a very similar expression patterns comparing with exons and introns with protein-coding genes (Figure 2c). Although lincRNAs are less conserved comparing to protein coding genes, their exons are significantly more conserved than introns (p < 0.0035) and random genome regions (p < 1.35e-14) (Figure 2d). These results provide strong evidence that the lincRNA is functional.

### Target prediction

We employed TargetScan [51] and PITA [52] to predict putative miRNA target sites on the exonic regions of lincRNAs by 7-mer and 8-mer seed matching (default parameters). We identified 44,887 putative binding sites that belong to 39,384 pairs of miRNA and lincRNA. All 525 lincRNAs and 677 miRNAs are involved in these predicted results. The same seed sequence could be shared in a miRNA family. A miRNA can also bind to multiple sites of a single lincRNA. We also downloaded conserved target prediction of miRNAs and genes for further analysis from the website of TargetScan, including 110,284 predicted pairs of 9,448 genes and 249 conserved human miRNAs.

### Expression reverse correlation

We quantified the expression levels of mRNA, lincRNA and precursor miRNAs using RPKM measures (reads per kilo-base exon model per million mappable reads). In total, there are 15,381 genes, 303 lincRNAs and 286 miRNAs expressed more than 0.5 RPKM in more than 15 samples. Our further analysis focuses on these sets of genes whose expression levels are detectable.

A generalized linear model (GLM) was used for modeling the potential effects of miRNA in down-regulating the expression levels of genes and lincRNAs in tumor and normal breast samples. Among the expressed mRNA, lincRNAs and miRNAs, 38,828 pairs are predicted to have regulatory relationships by miRNA target prediction algorithms. Among these potential target pairs, 1,742 and 213 mRNA-miRNA and lincRNA-miRNA pairs also showed significant reverse correlation, as derived from the GLM model (FDR<0.2).

**Figure 3:**
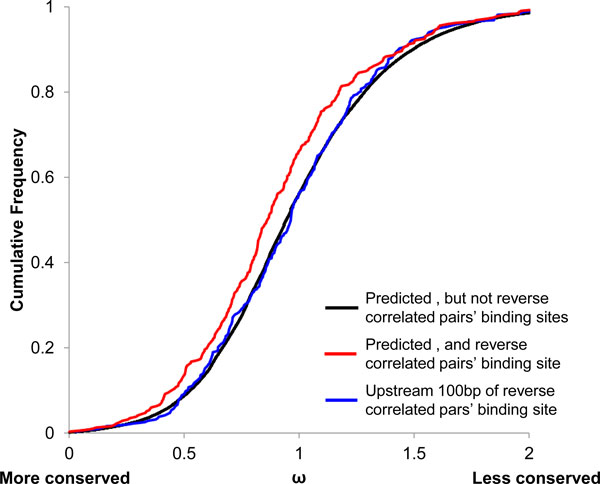
**Conservation score of predicted binding sites of miRNA binding sites on the lincRNAs**. X axis denotes ω  derived from Siphy prediction. Y axis denotes cumulative frequency. Red line is predicted seed match region for the lincRNA-miRNA pairs whose expression levels reversely correlated. Black line is predicted seed match region for the lincRNA-miRNA pairs whose expression levels do not show reversely correlation. Blue line is for the regions 100 bases upstream of predicted seed sites.

Among the 213 lincRNA-miRNA pairs, there are 315 predicted miRNA target sites (Additional file 2). Statistically, these sites are more conserved than other predicted target sites that did not show reverse correlation relationship (p < 7.24e-05, Wilcoxon test). They are also more conserved (p < 0.005, paired Wilcoxon test) than the regions 100bp upstream of the predicted target sites (Figure 3).

**Figure 4:**
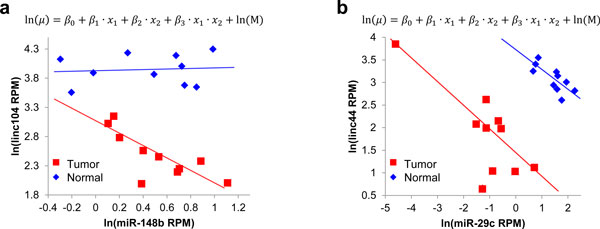
**MiRNA and lincRNA expression show different relationship between cancer and normal tissues**. X axis denotes miRNA expression. Y axis denotes lincRNA expression. Blue and red dots are for normal and tumor samples, respectively. (a) Hsa-miR-148b down-regulate linc104 in tumor samples, but not in normal samples. The coefficient of interaction item $\beta_{3}$ is significant. (b) hsa-miR-29c down-regulate linc44 in both normal and tumor samples. The coefficient of interaction item $\beta_{3}$ is not significant.

We further examined whether miRNAs showed difference in expression relationship with their potential target genes/lincRNAs in tumor and normal tissues. Of the predicted gene/lincRNA-miRNA pairs, 814 (~42%) show differences in expression relationships with a significant p-value for the GLM interaction factor (p < 0.05), including 121 (~57%) lincRNA-miRNA pairs (one example is shown in Figure 4a). Interestingly, only 336 pairs of gene-miRNA and 33 pairs of lincRNA-miRNA showed consistent regulatory relationship in both tumor and normal breast tissues (Figure 4b).

## Discussion

As a class of newly discovered non-coding RNA, genomic loci of lincRNAs are not well annotated. As an *ab initio *study, we inferred their sequences, strands, exon positions, and regulatory elements from RNA-seq data. At the current stage, the functions and regulatory mechanisms of most lincRNAs are unexplored. Several well-characterized lincRNAs are involved in the establishment of the chromatin state and it is believed that many lincRNAs execute their functions by closely associating with chromatin-modifying proteins [3]. This suggests trans-acting may be the primarily method for the affects of lincRNAs on gene expression [16].

Several recent studies revealed that thousands of lincRNAs exist in the cell [2,3,37,42]. As a transcript, the nature of lincRNAs are quite similar to mRNA, for instance, at least a fraction of lincRNAs are poly-adenylated and have exonic structures. This as evidence of the possibility that miRNA could bind to lincRNAs and trigger degradation. The repression of lincRNAs could be an unknown part of miRNA regulation. Since some lincRNAs have been observed to be differentially expressed in tumor cells compared to normal tissues [53], the miRNA-derived dysregulation of lincRNA expression can be another potential mechanism of tumorigenesis.

**Figure 5:**
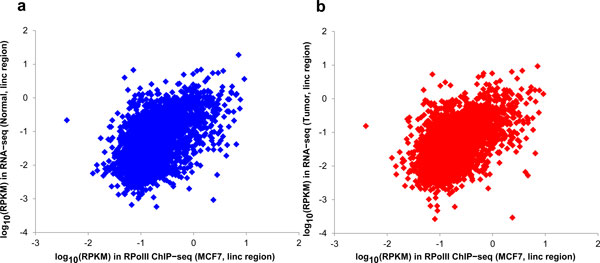
**Correlation between RPolII ChIP-seq signals in MCF7 cells and RNA-seq expression in breast tissues**. X axis denotes RPolII ChIP-seq signal level. Y axis denotes RNA-seq expression. (a) RNA-seq from normal breast tissue. (b) RNA-seq from breast tumor.

In addition to the data from RNA-seq experiments, two factors were considered for lincRNA identification, the transcriptional activity, and coding potential. ChIP-seq signals of RNA polymerase II (RPolII) were used to quantify transcription activity. One potential drawback of this strategy is that the RPolII data is from MCF7 cells due to the lack of measurement for the breast and tumor samples. This may cause bias in estimation. To ensure the RPolII data is representative of the transcript activity in breast, we surveyed the correlation between RPolII signal of MCF7 cell line and the RNA-seq levels of the breast tissues. We observed a very strong correlation (p < 6.8e-186, Spearman correlation) between RPolII signal in MCF7 cells and RNA-seq data from normal breast samples (Figure 5a~5b).

MiRNAs regulate degradation of its target mRNA through binding on the 3'-un-translated regions. Since the entire noncoding RNA is un-translated, the whole mature transcript can serve as potential miRNA target sites. We therefore predicted miRNA binding sites on the entire exonic regions of the identified lincRNAs.

One advantage of RNA-seq data is that it can simultaneously measure the expression level of both mRNAs/lincRNAs and precursor miRNAs in the same sample. This provides opportunity for revealing the relationship of the mRNA/lincRNA-miRNA in their expression levels. As a precursor stage of mature miRNA, pre-miRNA expression levels can be different from their final functional form. Therefore, major conclusions from this analysis are subject to further experimental validation.

Among 30,069 mRNA-miRNA pairs whose relationships were established based on target prediction alone, 31 pairs were experimentally validated, based on the records documented in the miR2Disease database and TarBase database [54,55]. Ten of these 31 (32.3%) pairs were among the 1,742 mRNA-miRNA pairs showed significant reverse correlation. Reported in 5 previous studies [56-60], these validated mRNA-miRNA pairs involve 5 miRNAs (hsa-miR-155, hsa-miR-29c, hsa-let-7b, hsa-miR-17, and hsa-miR-222) and 10 genes. In addition, we found many miRNAs showed difference in expression relationship with their potential target lincRNAs in tumor and normal tissues (Figure 4). This suggests that miRNA-lincRNA target can be specific to different cellular states.

## Methods

### RNA-seq data analysis

cDNA libraries from 10 normal breast tissues from the Susan G. Komen Tissue Bank at the IU Simon Cancer Center and 10 triple-negative breast cancer tumors were sequenced on an Applied Biosystems (ABI) SOLiD3 sequencer. We used a customized pipeline for RNA-seq data analysis, which includes three steps, QC filtering, sequence alignment, and gene expression quantification. 1) QC filtering. We first used SOLiD™ Instrument Control Software (ICS) and SOLiD™ Experiment Tracking System (SETS) software for the read quality recalibration. Sequences containing more than two 'N' or wildcards were discarded. Each sequence was scanned for low quality regions, and if a 5 base sliding window had an average quality score less than 20, the read would be truncated at that position. Any read with a length of less than 35 bases was discarded. Our experience suggests that such strategy effectively eliminates low quality reads while retaining high quality regions. 2) Alignment. We used BFAST [43] as our primary alignment algorithm, because it has high sensitivity for color space data [61]. We used a TopHat-like strategy [40] to align the sequencing reads that cross splicing junctions. After aligning the sequence reads to a filtering index including repeats, rRNAs (ribosomal RNA), and other sequences not of interest, we conduct sequence alignment on three levels: genomic, known junctions, and novel junctions (based on the enriched regions identified in the genomic alignment). 3) Gene expression level quantification. The expression levels of protein-coding genes and pre-miRNAs were quantified based on the total number of RNA-seq reads falling into their genomic regions. The data were further normalized as RPKM (reads per kilo-base exon model per million mappable reads) based on their length and sequencing depth.

In potential lincRNA regions, we systematically searched regions with short read enrichment as putative lincRNA exons. A putative exon must contain at least 8 reads. If the distance between two exons was less than 10 bases, the two exons would be merged together. To define the exact exon boundaries, we searched for potential splicing sites around the putative exons. Splicing donor/acceptor (GT-AG or CT-AC for RNAs on Watson or Crick strand respectively) sites within 25 bases from the 5'- and 3'- ends of two putative exons would be considered as possible splicing sites. A lincRNA junction library was constructed by connecting all the possible donor and acceptor sites within the 100,000 bases span. The minimum intronic length is set to be 70 bases. After aligning the remaining unmapped reads to the new lincRNA junction library, we used Scripture [42] for predicting the lincRNA exonic structures with default settings. Similar as for mRNAs and pre-miRNAs, the expression levels of lincRNAs were further quantified by the total number of sequencing reads falling into the lincRNA exonic regions. RPKM values were calculated for each lincRNA.

### Conservation, protein coding potential, and miRNA target prediction

We calculated the conservation score across placental mammals using Siphy [62] on various genomic regions, including exons and introns for protein coding genes, lincRNA exons and introns, random genomic regions, and predicted miRNA binding sites on lincRNAs. We used the ω  as the conservation score, which models the overall mutation rates across placental mammals [42]. The input of Siphy is a multiple alignment file (MAF) and a placental mammals model file. All known gene annotations (RefSeq), genome sequences (hg18 and mm9), liftOver tool, multiple alignment files, and placental mammals' model file were downloaded from the UCSC Genome browser.

We used the Codon Substantial Frequency (CSF) score to measure the coding potential of candidate lincRNA regions [49,50,63]. We set a 90 bases (30 Amino Acids) sliding window across the candidate lincRNA regions. The maximum score of any window of all six possible reading frames were used as the CSF score of the whole region [2]. A higher CSF score indicates higher potential for encoding protein-coding genes.

The mature and precursor miRNA annotations were extracted from miRBase [64]. The target prediction was conducted using the code downloaded from the TargetScan website [51].

### Generalized linear model, Wilcoxon test, and false discovery rate

We used a generalized linear model (GLM) to characterize the relationship between the expression levels of genes, lincRNAs and miRNAs, by treating gene/lincRNA and microRNA as dependent and independent variables, respectively. Assuming the number of the reads falling into a genomic region follows a Poisson distribution, a natural logarithm function was chosen as the link function:

(1) $$\text{ln}\left(y\right)=\beta_{0}+\beta_{1}\cdot x_{1}+\beta_{2}\cdot x_{2}+\beta_{3}\cdot x_{1}\cdot x_{2}+\text{ln}\left(M\right)+\varepsilon$$

In which y  denotes the number of reads that fall into the regions of gene/lincRNA; $x_{1}$ denotes pre-miRNA expression, represented by the natural logarithm value of reads number normalized by total mapped reads, ln(RPM); $x_{2}$ is a categorical variable having two possible values, indicating normal or tumor status; $x_{1}\cdot x_{2}$ is an interaction factor that represents the influence of the biological conditions to miRNA regulation patterns; ln(M) is the natural logarithm value of the total reads number as an offset; $\beta_{0},...,\beta_{3}$ are intercept and slopes of the predictors and their combinations; ε  denotes the variation that cannot be explained by the independent factors.

To allow over-dispersion, in practice, we employed a quasi-*Poisson *distribution. For the significance tests on the global gene/lincRNA-miRNA expression correlation, we employed a t-test on the miRNA expression coefficient $\beta_{1}$. If a gene/lincRNA-miRNA pair did not show significance for $\beta_{3}$, which means the relationship between miRNA and its target is not affected by the cellular states (tumor or normal), we conducted GLM model by removing the interaction term.

To correct for multiple hypothesis testing, false discovery rate (FDR) was calculated based on Benjamini-Hochberg correction [65]. All statistical models and tests (GLM, Wilcoxon test, Chi-square test) were implemented in R: http://www.r-project.org/.

## Competing interests

The authors declare that they have no competing interests.

## Authors' contributions

LJ, YW, and YL contributed to the design of the study. LJ and YL designed and performed the computational modeling and drafted the manuscript. GW, SC and YW participated in coordination, discussions related to result interpretation and revision of the manuscript. MR, BS and SC contributed to the experiment performing and data collection. All the authors read and approved the final manuscript.

## Supplementary Material

Additional file 1 **Genomic coordinates of 525 lincRNAs showed expression evidence in breast tissues**. Click here for file

Additional file 2 **Genomic coordinates of the 315 predicted microRNA binding sites on lincRNAs**. Click here for file
